# High quality and quantity Genome-wide germline genotypes from FFPE normal tissue

**DOI:** 10.1186/1756-0500-4-159

**Published:** 2011-05-26

**Authors:** Lisa A Cannon-Albright, Kendal G Cooper, Ann Georgelas, Philip S Bernard

**Affiliations:** 1 Genetic Epidemiology, Department of Internal Medicine, University of Utah School of Medicine, Salt Lake City, UT, USA; 2 George E. Wahlen Department of Veterans Affairs Medical Center, Salt Lake City, Utah, USA; 3 Tissue Resource and Applications Core, Huntsman Cancer Institute, University of Utah, Salt Lake City, UT, USA; 4 Department of Pathology, University of Utah Health Sciences Center, Salt Lake City, UT, USA; 5 The ARUP Institute for Clinical and Experimental Pathology, SLC, Utah, USA

## Abstract

**Background:**

Although collections of formalin fixed paraffin embedded (FFPE) samples exist, sometimes representing decades of stored samples, they have not typically been utilized to their full potential. Normal tissue from such samples would be extremely valuable for generation of genotype data for individuals who cannot otherwise provide a DNA sample.

**Findings:**

We extracted DNA from normal tissue identified in FFPE tissue blocks from prostate surgery and obtained complete genome wide genotype data for over 500,000 SNP markers for these samples, and for DNA extracted from whole blood for 2 of the cases, for comparison.

Four of the five FFPE samples of varying age and amount of tissue had identifiable normal tissue. We obtained good quality genotype data for between 89 and 99% of all SNP markers for the 4 samples from FFPE. Concordance rates of over 99% were observed for the 2 samples with DNA from both FFPE and from whole blood.

**Conclusions:**

DNA extracted from normal FFPE tissue provides excellent quality and quantity genome-wide genotyping data representing germline DNA, sufficient for both linkage and association analyses. This allows genetic analysis of informative individuals who are no longer available for sampling in genetic studies.

## Findings

Our interest in exploring a wider utility for stored FFPE tissue began with the desire for genome-wide (GW) genotyping data for individuals who would be informative to a linkage study in a high-risk pedigree, but who were not available for sampling. Recognizing that normal tissue is typically included in FFPE samples (in addition to tumor tissue), we hypothesized that DNA could be extracted from normal tissue found in most FFPE samples, and that this would provide adequate genome-wide germline DNA for genotyping.

It is recognized that the processes of fixation and storage of FFPE samples lead to degradation and base modification with chemical residues. FFPE-extracted DNA (for tumor tissue) has been reported to be adequate for sequencing, directed genotyping and RNA extraction, but it has been reported that only partial data are typically obtained for genome-wide genotyping. Available genotyping platforms report poor performance of GW genotyping data for DNA extracted from FFPE tumor samples; and we could not find published evidence for genome-wide genotyping results for DNA extracted from FFPE normal tissue samples.

Even poor genotyping yields for DNA extracted from FFPE would provide adequate data for genetic linkage studies. Many fewer markers are necessary for genome-wide linkage analysis than for genome-wide association analysis (GWAS). For linkage analysis, markers in linkage disequilibrium (LD) are not used (due to introduction of bias), and a much smaller subset of SNP markers can represent the genome. Illumina's original genome-wide SNP set for linkage consisted of 6,000 SNPs. We have previously performed successful genome-wide linkage using subsets of SNPs with no LD from the Illumina 550k SNP data set. We selected 27,157 markers representing the entire genome; median heterozygosity was 0.49 and median spacing was 0.14 cM [1].

## Methods

We identified 5 prostate cancer cases with FFPE tissue samples stored between 18-41 years ago in different facilities (Table 1). A 5-micron cut was made from each FFPE tissue block and stained with hematoxylin and eosin (H&E) for histological review. Tumor rich areas were circled on the H&E slide and aligned with the original tissue block; full face cuts (4 × 10 micron) were made from blocks containing no tumor tissue to capture grossly uninvolved (i.e. "normal") tissue. There was sufficient normal tissue in 4 of these 5 samples; the oldest sample did not have sufficient normal tissue for extraction (case 423197 in Table 1).

**Table 1:** Summary of the 5 tissue block samples tested.

Case Sample	year	#blocks/slides	DNA yield(μg)	% SNP genotype
423197	1969	3	*no normal tissue	NA
525593	1984	14	48	.891
620691	1985	3	6	.971
723932	1990	8	12	.989
826190	1992	1/1	5	.873

Tissue punches from normal tissue were combined and de-paraffinized in Citri Solv followed by dehydration in 100% Ethanol. DNA was isolated from the dried tissue using the Qiagen QIAamp DNA FFPE Tissue Kit. The yield, purity and integrity of DNA samples was assessed using spectrophotometry and gel electrophoresis. Both yield and purity was excellent, with at least 5 μg obtained from each sample and A260/280 ratios of 1.8. However, the integrity of the DNA was questionable, with significant fragmentation detectable upon electrophoresis (data not shown).

We provided DNA samples for 4 cases with normal tissue extracted to deCODE Genetics for genome-wide genotyping with the 610Q platform. For one of these 4 cases (723932), we also provided deCODE with a DNA sample which was extracted from whole blood. For another case with an FFPE extracted sample (826190) we had previously received GW genotyping from deCODE for a DNA sample extracted from whole blood. For these two-paired samples of DNA from FFPE normal and DNA from whole blood from the same individuals we estimated reliability. This research was approved by the University of Utah IRB, and all subjects were appropriately consented.

## Results and Discussion

Table 1 summarizes the 5 tissue samples considered (the first of which yielded no normal DNA). The genotyping call rates for the 4 DNA case samples from FFPE ranged from 0.873 to 0.989. For the case for which we submitted both DNA from whole blood and also DNA from normal tissue from FFPE (marker yield = 0.999) at the same time (723932), we observed 0.9998 reproducibility in genotyping calls. For one of the other cases with an FFPE sample (826190) we had obtained GW genotyping results previously; concordance between the 2 samples was 0.993. The only slightly less than 100% concordance may be due to drop outs or may represent the very low genotyping error rates observed for SNP arrays; such a low error rate is likely to have little or no impact on identity-by descent inference for disease linkage. These surprisingly high success rates for number of calls and for reproducibility of calls for DNA extracted from FFPE may be the norm, rather than the exception, as these 4 samples were chosen randomly and represent a range of FFPE tissue block quality, quantity and age.

Previous studies have illustrated that DNA present within FFPE tissue blocks can be used for molecular studies [2-7]. However, these previous studies have primarily focused on CNV LOH studies, and small numbers of SNPs; they did not perform or compare genome-wide genotyping results for normal tissue,. There is significant variability in how FFPE samples are collected, processed, and stored [8]. Factors that affect the quality of nucleic acids within FFPE tissue blocks include the time from de-vascularization to fixation, the method of fixation, and the age of the tissue block [8]. While pre-analytical testing can provide some information about the suitability of a sample for downstream molecular applications ([9], it is often unknown whether a given technology can yield useful data from a sample until the final analyses.

## Conclusions

Our attempt to generate genome-wide genotyping data for individuals not available for genotyping, has been both simple and revolutionary in several ways. We have shown that "normal" tissue from archived FFPE samples (even decades old) is an excellent source of germline DNA for individuals no longer available for sampling. We have also shown that both high quality and high yield genome-wide genotyping data can be obtained from DNA extracted from normal FFPE tissue (even when samples are stored long term). Finally, we propose that the GW genotyping data from DNA extracted from normal tissue from FFPE samples is more than adequate to allow GW linkage analysis despite significant fragmentation, and might be considered adequate for GW association studies (with some modifications to methods, given the slightly smaller number of SNPs available).

In Utah, as an example, the largest health care provider has wisely stored all pathology samples available since the 1960s. For such long-standing bio-repositories, the methods we propose could be important for the study of disorders which are no longer commonly diagnosed worldwide, but which still have important lessons to teach, and for which stored samples are available, e.g. pandemic influenza. These findings are especially important for disorders for which there are few living cases (e.g. disorders with short survival times).

We are aware that some companies have developed additional protocols designed to improve yield of genotyping data from DNA extracted from FFPE samples; this will further enhance the value of stored FFPE samples. Our innovative solution to procuring genome-wide genotyping data for informative individuals not otherwise available for sampling has provided a powerful way forward for a number of different genetic studies which require genome-wide genotyping data of good quality and quantity. Those researchers and facilities with the foresight to store FFPE tissue have at their fingertips a vastly informative and valuable resource that we propose will allow many investigations not previously thought possible.

## List of Abbreviations

FFPE: formalin fixed paraffin embedded; SNPs: single nucleotide polymorphism.

## Competing interests

The authors declare that they have no competing interests.

## Authors' contributions

The project was conceived of by LACA who also provided tissue; PB performed the pathologic examination and selection of sample tissue; KC performed the DNA extraction under the direction of AG; all authors contributed to study design and manuscript revisions. All authors read and approved the final manuscript.

## References

[B1] Allen-Brady K Norton PA Farnham JM Teerlink C Cannon-Albright LA Significant Linkage Evidence for a Predisposition Gene for Pelvic Floor Disorders on Chromosome 9q21 Am J Hum Genet 2009 84 5 678 82 10.1016/j.ajhg.2009.04.002 19393595 PMC2681003

[B2] Gnanapragasam VJ Unlocking the molecular archive: the emerging use of formalin-fixed paraffin-embeddeed tissue for biomarker research in urological cancer Br J Urol International 2009 105 274 278 10.1111/j.1464-410X.2009.08665.x 19519763

[B3] Oosting J Lips EH van Ejik R Eilers PH Szubhai K Wijmenga C Morreau H van Wezel T High-resolution copy number analysis of paraffin-embedded archival tissue using SNP BeadArrays Genome Res 2007 17 3 368 76 Epub 2007 Jan 31 10.1101/gr.5686107 17267813 PMC1800928

[B4] Hennig G Gehrmann M Stropp U Brauch H Fritz P Eichelbaum M Schwab M Schroth W Automated extraction of DNA and RNA from a single formalin-fixed paraffin-embedded tissue section for analysis of both single-nucleotide polymorphisms and mRNA expression Clin Chem 2010 56 12 1845 53 10.1373/clinchem.2010.151233 20947696

[B5] Sikora M Thibert JN Salter J Dowsett M Johnson MD Rae JM High-efficiency genotype analysis from formalin-fixed paraffin-embedded tumor tissues Pharmacogenomics J 2010 in press 10.1038/tpj.2010.50 PMC2996486 20548328

[B6] Thompson ER Herbertt SC Forrest SM Camplbell IG Whole genome SNP arrays using DNA derived from formalin-fixed, paraffin-embedded ovarian tumor tissue Hum Mutat 2005 26 4 384 9 10.1002/humu.20220 16116623

[B7] Reid AH Fanning TG Hultin JV Taubenberger JK Origin and evolution of the 1918 "Spanish" influenza virus hemagglutinin gene Proc Natl Acad Sci 1999 96 1651 56 10.1073/pnas.96.4.1651 9990079 PMC15547

[B8] Hewitt SM Lewis FA Yanxiang C Conrad RC Cronin M Danenberg KD Goralski TJ Langmore JP Tissue Handling and Specimen Preparation in Surgical Pathology: Issues Concerning the Recovery of Nucleic Acids from Formalin-Fixed, Paraffin-Embedded Tissue Arch Pathol Lab Med 2008 132 1929 1936 19061293 10.5858/132.12.1929

[B9] Panaro NJ Yuen PK Sakazume T Fortina P Kricka LJ Wilding P Evaluation of DNA fragment sizing and quantification by the agilent 2100 bioanalyzer Clin Chem 2000 46 1851 1853 11067828

